# The causes of Fanconi anemia in South Asia and the Middle East: A case series and review of the literature

**DOI:** 10.1002/mgg3.1693

**Published:** 2021-05-07

**Authors:** Ashley S. Thompson, Nusrat Saba, Lisa J. McReynolds, Saeeda Munir, Parvez Ahmed, Sumaira Sajjad, Kristine Jones, Meredith Yeager, Frank X. Donovan, Settara C. Chandrasekharappa, Blanche P. Alter, Sharon A. Savage, Sadia Rehman

**Affiliations:** ^1^ Clinical Genetics Branch Division of Cancer Epidemiology and Genetics National Cancer Institute Bethesda MD USA; ^2^ Institute of Biomedical and Genetic Engineering Islamabad Pakistan; ^3^ Quaid‐i‐Azam International Hospital Islamabad Pakistan; ^4^ Cancer Genomics Research Laboratory Leidos Biomedical Research Frederick National Laboratory for Cancer Research Frederick MD 20850 USA; ^5^ Cancer Genetics and Comparative Genomics Branch National Human Genome Research Institute National Institutes of Health Bethesda MD USA

**Keywords:** Fanconi anemia, genetic testing, hematopoietic cell transplantation, inherited bone marrow failure syndrome, population genetics

## Abstract

**Background:**

Fanconi anemia (FA) is an inherited bone marrow failure syndrome associated with characteristic dysmorphology primarily caused by biallelic pathogenic germline variants in any of 22 different DNA repair genes. There are limited data on the specific molecular causes of FA in different ethnic groups.

**Methods:**

We performed exome sequencing and copy number variant analyses on 19 patients with FA from 17 families undergoing hematopoietic cell transplantation evaluation in Pakistan. The scientific literature was reviewed, and we curated germline variants reported in patients with FA from South Asia and the Middle East.

**Results:**

The genetic causes of FA were identified in 14 of the 17 families: seven *FANCA*, two *FANCC*, one *FANCF*, two *FANCG*, and two *FANCL*. Homozygous and compound heterozygous variants were present in 12 and two families, respectively. Nine families carried variants previously reported as pathogenic, including two families with the South Asian *FANCL* founder variant. We also identified five novel likely deleterious variants in *FANCA*, *FANCF*, and *FANCG* in affected patients.

**Conclusions:**

Our study supports the importance of determining the genomic landscape of FA in diverse populations, in order to improve understanding of FA etiology and assist in the counseling of families.

## Graphical abstract

Fanconi anemia (FA) primarily caused by biallelic pathogenic germline variants in any of 22 different DNA repair genes but there are limited data on the specific FA gene variants in cases from the Middle East and South Asia. We identified novel and known genetic causes of FA in 14 of 17 families from Pakistan. Our study supports the importance of determining the genomic landscape of FA in diverse populations, in order to improve understanding of FA etiology and assist in the counseling of families.

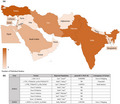

## 1 INTRODUCTION

Fanconi anemia (FA, MIM:227650) is a cancer‐prone inherited bone marrow failure syndrome associated with radial ray abnormalities, characteristic facies, and other medical problems (Fiesco‐Roa et al., 2019; Kottemann & Smogorzewska, 2013). Approximately 5% of patients with FA have the VACTERL‐H phenotype (Vertebral anomalies, Anal atresia, Cardiac anomalies, Tracheoesophageal fistula, Esophageal atresia, Renal structural anomalies, Limb anomalies [primarily radii and/or thumbs], and Hydrocephalus; Alter & Rosenberg, 2013). Additional FA phenotypic findings are associated with Pigmentation of the skin, small Head, small Eyes, central Nervous system anomalies (excluding hydrocephalus), Otologic anomalies, and Short stature (PHENOS; Alter & Giri, 2016). Patients with FA have exceedingly high risks of head and neck squamous cell carcinoma (HNSCC) and leukemia compared with the general population (Alter et al., 2018). FA‐associated bone marrow failure (BMF) frequently requires hematopoietic cell transplantation (HCT).

The laboratory diagnosis of FA is based on increased chromosome breakage in lymphocytes or fibroblasts after culture with clastogens such as mitomycin C (MMC) or diepoxybutane (DEB) and can be confirmed by germline genetic testing (Fargo et al., 2014). The majority of individuals with FA have autosomal recessive inheritance of pathogenic germline variants in specific DNA repair genes (Fiesco‐Roa et al., 2019; Knies et al., 2017). *FANCB* and *FANCO/RAD51C* are the exceptions, inherited in an X‐linked and autosomal dominant pattern, respectively. *FANCA* accounts for approximately 65% of cases, while *FANCC* and *FANCG* account for an additional 20% of cases in individuals of European ancestry (Wang & Smogorzewska, 2015). FANCA, FANCB, FANCC, FANCE, FANCF, FANCG, FANCL, and FANCM comprise the core FA complex (upstream) in conjunction with FANCT/UBE2T, which is responsible for recognizing interstrand crosslinks and activating the FA DNA repair pathway through interaction with FANCD2 and FANCI (the ID complex). The downstream pathway facilitates DNA repair by homologous recombination performed by FANCD1/BRCA2, FANCJ/BRIP1, FANCN/PALB2, FANCO/RAD51C, FANCP/SLX4, FANCQ/ERCC4, FANCR/RAD51, FANCS/BRCA1, FANCU/XRCC1, FANCV/REV7, and FANCW/RFWD3 (Inano et al., 2017; Kitao & Takata, 2011; Rodriguez & D'Andrea, 2017).

There have been a limited number of reports on the genetic etiology of FA in populations from South Asia and the Middle East. Such studies have identified several novel disease‐causing germline genetic variants, including the first reports of FA caused by pathogenic variants in *FANCO*/*RAD51C* or *FANCE* (Aftab et al., 2017; Aslan et al., 2015; Aymun et al., 2017; Balta et al., 2000; Castella et al., 2011; de Winter et al., 2000; Donovan et al., 2019; Dorsman et al., 2007; Esmail Nia et al., 2016; Ghazwani et al., 2016; Gille et al., 2012; Kalb et al., 2007; Koc et al., 1999; Levran et al., 1997; Moghadam et al., 2016; Salem et al., 2014; Shahid et al., 2019; Shamseldin et al., 2012; Shukla et al., 2013; Solanki et al., ,^2016^, ^2017^; Tamary et al., ,^2000^, ^2004^; Vaz et al., 2010; Vundinti, 2014; Waisfisz et al., 1999; Wegner et al., 1996; Wijker et al., 1999; Zareifar et al., 2019) as well as discovery of a founder mutation in *FANCL* (Donovan et al., 2019), highlighting the importance of germline genetic studies of FA in underrepresented regions. In this report, we evaluated the genetic causes of FA in 19 patients from 17 unrelated families being considered for HCT in Pakistan and conducted a detail review of the causes of FA in South Asia and the Middle East.

## 2 METHODS

### 2.1 Editorial policies and ethical compliance

This project was approved by the ethical review committee of the Institute of Biomedical and Genetic Engineering (IBGE, Islamabad, Pakistan). Written informed consent was obtained from all study participants or the parent or guardian of participants who were under the age of 18 years old. Consent for the publication of identifying images or other personal or clinical details of participants that have the potential to compromise anonymity was obtained from all study participants or the parent or guardian of participants who were under the age of 18 years old.

### 2.2 Study subjects

Individuals with FA and their first‐degree relatives were evaluated by their referring physicians. Chromosome breakage using MMC on primary lymphocytes was performed at the Armed Forces Institute of Bone Marrow Transplant Center (ABMTC, Rawalpindi, Pakistan; Cervenka et al., 1981). All affected individuals had chromosome breakage results consistent with FA and severe BMF necessitating evaluation for HCT. De‐identified blood‐derived DNA samples from 17 unrelated families, including 19 individuals with FA and 33 relatives, were sent to the National Cancer Institute's Cancer Genomics Research Laboratory (NCI CGR, Gaithersburg, MD, USA) for sequencing and genotyping; a list of all study participants and their disease status is in Table S1.

### 2.3 Sequence analysis

Exome sequencing was performed at NCI CGR as previously described (Ballew et al., 2013). Variants were filtered based on their presence as homozygous or biallelic in previously identified FA‐associated genes. Data from family members was evaluated when available. Variant annotation was performed using ANNOVAR (Wang et al., 2010) and the computational resources of the National Institutes of Health (NIH) High Performance Computing (HPC) Biowulf cluster (http://hpc.nih.gov). Additional filters applied include a Genome Aggregation Database (gnomAD) minor allele frequency (MAF) <1% in all populations, bioinformatic prediction tools (criteria for deleterious variants: MetaSVM >0, REVEL ≥0.5, and CADD phred >20), and clinical significance indicators (ClinVar and InterVar; Ioannidis et al., 2016; Karczewski et al., 2019; Kim et al., 2017; Kircher et al., 2014; Landrum et al., 2018; Li & Wang, 2017). Potential splice site variants were assessed using Human Splicing Finder (Desmet et al., 2009). We used BAM‐matcher and vcftools to assess potential consanguinity as described (Manichaikul et al., 2010; Wang et al., 2016; Yang et al., 2010).

Copy number variations (CNV) were detected using VarSeq™ v2.1 (VS‐CNV), which analyzes changes in WES coverage between the sample and controls (Fortier et al., 2018), and the detected CNVs were visually evaluated using GenomeBrowse® (Golden Helix, Inc.; Golden Helix, 2018a, 2018b). Homozygous or heterozygous deletions were filtered based on p‐values (< 0.001) and annotated using ClinVar (Landrum et al., 2018). Z‐scores and ratio values were assessed when validating genotypes. Suspected CNV events underwent validation using targeted whole gene sequencing as described (Chandrasekharappa et al., 2013).

DNA from families 3‐FA, 8‐FA, 9‐FA, 12‐FA, 14‐FA, and 16‐FA was also sequenced using a targeted custom capture design (Roche, Inc.) for next generation sequencing (NGS) designed to include all known FA genes and FA candidate genes, including all intronic regions and 5 kb upstream and downstream of each gene, as previously described (Chandrasekharappa et al., 2013). Sequence reads were aligned to human genome build 19 (GRCh37) using Burrows‐Wheeler Alignment tool and variants were called using HaplotypeCaller and the GATK best practices pipeline for germline variants (DePristo et al., 2011; H. Li & Durbin, 2009; Van der Auwera et al., 2013). SNVs and indels were annotated using ANNOVAR (K. Wang et al., 2010) CNVs were detected from the targeted NGS reads and annotated using Nexus Copy Number version 10.0 (BioDiscovery, Inc.).

Additionally, DNA from family 17‐FA was sequenced using PacBio^®^ long‐range sequencing technology with custom IDT xGen^®^ Lockdown^®^ Probes designed to capture all intronic and exonic regions of *FANCA*. The manufacturer's PacBio^®^ protocols were followed for shearing genomic DNA, end repair, ligation of linear barcoded adapters, amplification, sample pooling, and capturing using IDT xGen^®^ Lockdown^®^ Probes. Libraries were prepared using SMRTbell^®^ protocol for primer annealing, polymerase binding, and sequencing on the Sequel system. Circular consensus reads were generated using default parameters (3 passes, 0.99 accuracy) and demultiplexed according to parameters for symmetrical barcodes. Sequence reads were aligned to human genome build 19 (GRCh37) and structural variants were called using pbsv default parameters.

### 2.4 Compilation of FA gene variants reported in South Asia and the Middle East

A comprehensive literature review was performed using the The National Library of Medicine's PubMed database using the following search terms combined with countries in South Asia and the Middle East, (e.g. “Fanconi anemia and Pakistan” or “Fanconi and India”) to curate previously published FA gene variants reported in patients from the following regions (accessed July 10, 2020): Afghanistan, Bangladesh, Egypt, India, Iran, Iraq, Israel, Jordan, Lebanon, Nepal, Oman, Pakistan, Saudi Arabia, Syria, Turkey, and Yemen. Large cohort studies and case reports which only consisted of phenotypic data and did not report patients’ specific genotypes were excluded. Studies which only reported FA subtypes by complementation testing were also excluded unless further sequencing efforts revealed the specific variant(s) in the patient(s).

## 3 RESULTS

### 3.1 Participant characteristics

**FIGURE 1:**
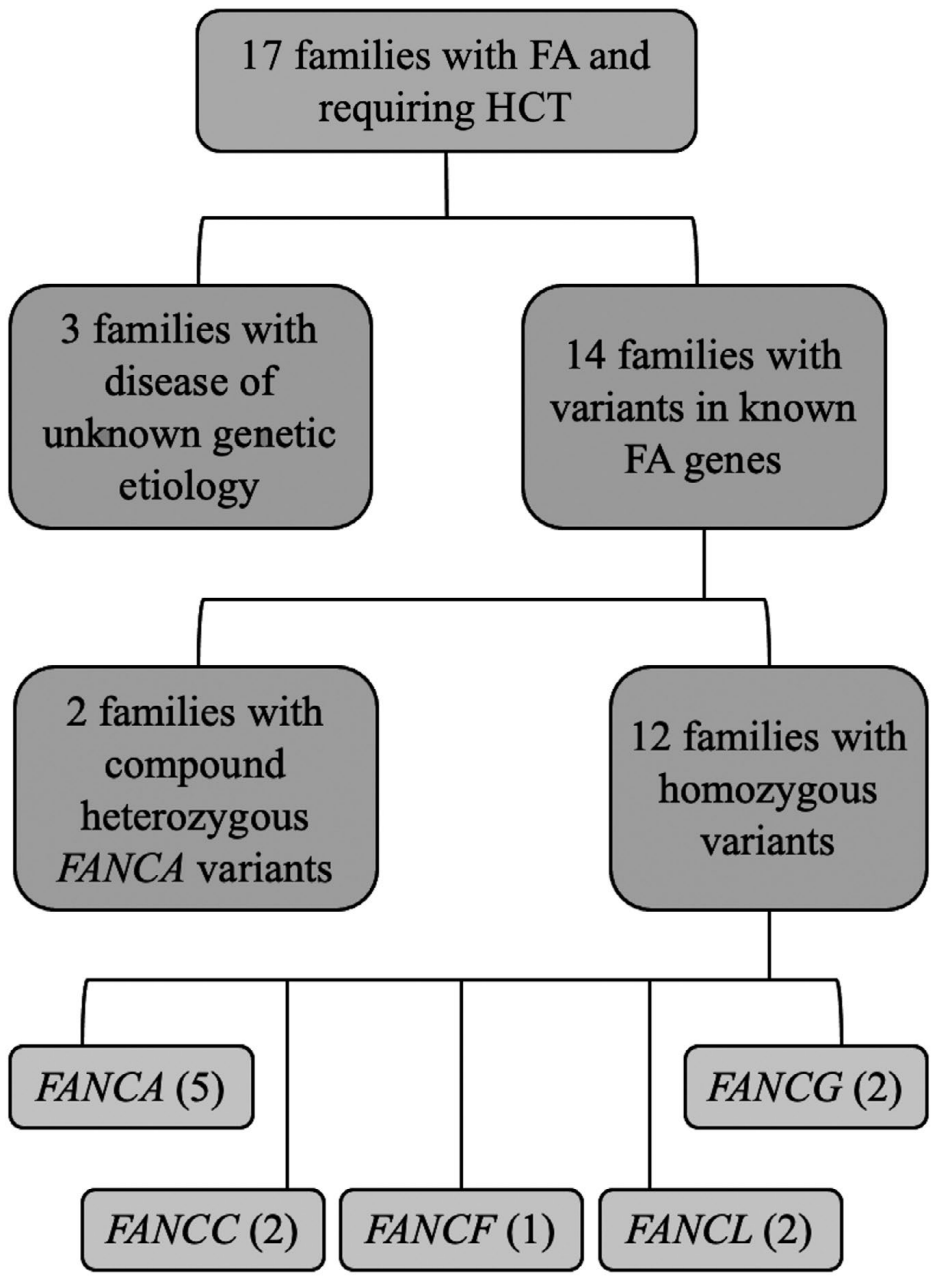
A flowchart of the genetic findings in 19 patients with FA from 17 unrelated families evaluated in this study

There were 19 patients with FA (16 males and 3 females) from 17 unrelated families evaluated in this study (Figure 1). The majority of families (12/17, 70%) were from Northern or Central Punjab. Other families were from Southern Punjab, Islamabad, Khyber Pakhtunkhwa, and Azad Kashmir. The median age at FA diagnosis was 7 years (range 4–12), and while all were evaluated for HCT, only 5 patients underwent matched sibling HCT. Eight of the 19 participants were deceased at the time of this study. The median age at death was 8.5 years (range 4–13). Pathogenic variants relevant to FA were identified in 14 families with *FANCA* being the most common (7/14, 50%). Homozygous variants in FA‐associated genes were identified in 12 of the 14 solved families (86%) and 2 probands had compound heterozygous variants. Physical and genetic findings for all participants are listed in Tables 1 and 2, respectively.

**TABLE 1:** Clinical features of study participants with Fanconi anemia.

Patient ID^a^	Sex	Age at Diagnosis (Years), Vital Status	Age at and Cause of Death	Skin	Hand	Renal	HCT (Yes/No)^b^	Other
1‐FA	M	7, deceased	9, NR			Ectopic kidneys	No	
3‐FA	M	5, alive	—	Café au lait macules	Thumb malformation		Yes	Short stature
4‐FA	M	7, alive	—				Yes	
5‐FA	M	11, deceased	12, NR				No	Abnormal left leg growth, short stature
6‐FA	M	9, deceased	10, hemorrhage NOS	Hyperpigmentation	Polydactyly		No^c^	
7‐FA	M	7, deceased	8, NR					
8‐FA	M	4, deceased	4, brain hemorrhage		Absent radii and metacarpal bones		No^c^	High arched palate, skeletal malformations NOS
9‐FA	F	10, alive	—				Yes	
10‐FA	M	5, deceased	6, hemorrhagic stroke		Polydactyly		No	
12‐FA	M	11, alive	—				Yes	
14‐FA	M	12, deceased	13, NR				No	
16‐FA	F	9, alive	—		Bilateral thumb malformations		Yes	
17_01‐FA	M	6, alive	—		Thumb malformation		No	Immunodeficiency
17_02‐FA	M	10, alive	—		Thumb malformation		No	Immunodeficiency
18‐FA	M	5, alive	—		Polydactyly and small right hand	Ectopic kidneys	Yes	Short stature
19‐FA	F	8, unknown	—				No	
20‐FA	M	5, alive	—				No	
21_01‐FA	M	8, deceased	13, NR				Yes	Numerous rosettes with central eosinophilic material on bone marrow biopsy
21_02‐FA	M	5, alive	—					Numerous rosettes with central eosinophilic material on bone marrow biopsy

Abbreviations: F, Female; HCT, hematopoietic cell transplantation; M, Male; NOS, not otherwise specified; NR, Not reported.

^a^ All participants had bone marrow failure and were being considered or underwent hematopoietic cell transplantation. Empty boxes indicate where clinical information was not available.

^b^ All participants who successfully underwent HCT received material from an HLA‐matched sibling. Participants who did not undergo transplant did not have a matched donor, unless indicated otherwise.

^c^ Participant died prior to HLA‐matched sibling HCT.

**TABLE 2:** Germline genetic variants identified in study participants with Fanconi anemia

Gene	Patient ID	Genotype	Variant	Previously reported	ClinVar	gnomAD % MAF^a^ All; South Asian ancestry (Karczewski et al., 2019)	Consequence of Variant^b^ (Ioannidis et al., 2016; Kim et al., 2017; Kircher et al., 2014)
*FANCA*	1‐FA	Homozygous	c.3788_3790delTCT, p.Phe1263del	Castella et al. (2011)	Pathogenic	0.009929; 0	In‐frame deletion
	3‐FA	Compound Heterozygous	g.89871674‐89880557del	(Solanki et al., 2016)	NR		Exons 4‐7 Deletion
			g.89861527‐89863726del		NR		Exon 11 Deletion
	4‐FA	Homozygous	c.37dupC, p.Gln13Profs*24		NR		Frameshift
	9‐FA	Homozygous	g.89856782‐89874222del	Esmail Nia et al. (2016)	NR		Exons 7‐14 Deletion
	17_01‐FA and 17_02‐FA	Compound Heterozygous	c.2749C>T, p.Arg917*	Solanki et al. (2016)	Pathogenic		Stop‐gain
			g.89847600‐89853759del	Gille et al. (2012)	NR		Exons 15‐17 Deletion
	19‐FA	Homozygous	c.4070C>A, p.Ala1357Asp		NR		Missense, MetaSVM 0.9149, REVEL 0.702, CADD phred 24.1
	20‐FA	Homozygous	c.1541C>A, p.Ala514Asp		NR	**0.000398; 0.003266**	Missense, MetaSVM 0.876, REVEL 0.851, CADD phred 27.1
*FANCC*	5‐FA	Homozygous	c.1642C>T, p.Arg548*	Aftab et al. (2017)	Pathogenic	0.004954; 0.01307	Stop‐gain
	8‐FA	Homozygous	c.1642C>T, p.Arg548*				
*FANCF*	10‐FA	Homozygous	c.785T>G, p.Leu262*		NR	0.0007955; 0.003266	Stop‐gain
*FANCG*	18‐FA	Homozygous	c.710C>G, p.Ser237*		NR		Stop‐gain
	21_01‐FA and 21_02‐FA	Homozygous	c.1471_1473delAAAinsG, p.Lys491Glyfs*9	Gille et al. (2012)	NR	**0.0003976; 0.003266**	Frameshift
*FANCL*	6‐FA	Homozygous	c.1092G>A, p.Trp341_Lys364del	Donovan et al. (2019)	NR	**0.001994; 0.01634**	Exon 13 Skipping
	7‐FA	Homozygous	c.1092G>A, p.Trp341_Lys364del				

Abbreviations: MAF, minor allele frequency; NR, Not reported.

*in silico* tools were used to predict the pathogenicity of missense variants.

^a^ % MAF in bold indicate these variants were only reported in South Asian populations. Blank cells indicate the variant was not present in gnomAD.

### 3.2 *FANCA* variants and phenotypes

Individual 1‐FA presented at age 7 years with aplastic anemia which progressed to severe BMF. He had ectopic kidneys, but no other phenotypic features were reported. We identified a homozygous in‐frame deletion in exon 38 of *FANCA* (c.3788_3790delTCT, p.Phe1263del, NC_000016.9:g.89807250_89807252delAGA, rs397507553, ClinVar:41003). Both parents were heterozygous carriers and his sibling was wild‐type. The c.3788_3790delTCT variant is the most frequently reported *FANCA* variant and has been observed in multiple populations throughout the world, including FA patients from Pakistan (Castella et al., 2011; Kimble et al., 2018; Shahid et al., 2019). This in‐frame deletion variant has particularly high prevalence in Spain and Brazil at an allele frequency of 0.01696% and 0.01633% in Latino/Admixed American and non‐Finnish European populations, respectively (Castella et al., 2011; Levran et al., 1997).

**FIGURE 2:**
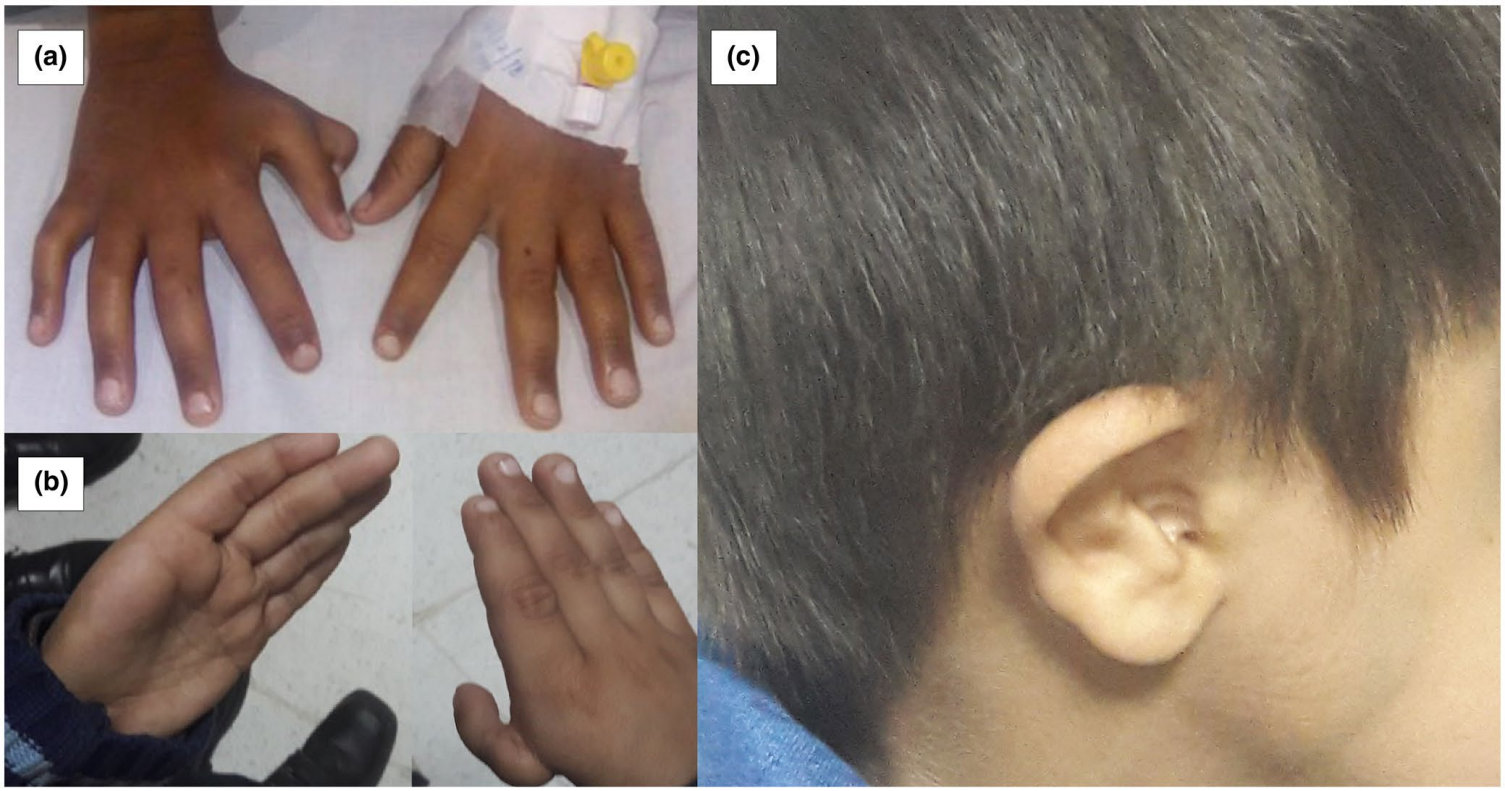
(a) An abnormal right thumb noted at birth in 3‐FA, (b) an absent left thumb and abnormal right thumb in patient 17_01‐FA, and (c) a small ear canal seen in Patient 17_01‐FA

Individual 3‐FA had an abnormal thumb (Figure 2a) and café au lait spots noted at birth, as well as short stature and low gonadotrophin hormone levels. FA was diagnosed by chromosome breakage on primary lymphocytes after he presented at 5 years of age with neutropenia that progressed to severe BMF. He underwent successful HLA‐matched sibling donor HCT at the age of 6 years. 3‐FA had two deletions in *FANCA* (NC_000016.9:g.89871674_89880557del, and NC_000016.9:89861527_89863726del) affecting exons 4–7 and 11, respectively. These two deletions have been previously reported in an Indian FA patient (Solanki et al., 2016). The exon 11 deletion was paternally inherited, while the deletion of exons 4–7 was maternally inherited. Validation by targeted sequencing methods determined that one unaffected sibling did not carry either deletion. Another unaffected sibling was predicted to be a carrier of the exon 4–7 deletion by VS‐CNV but there was insufficient DNA for sequencing validation.

Severe BMF developed at 7 years of age in individual 4‐FA. Hemophagocytosis was reported on his bone marrow biopsy but no other phenotypic information was available. He underwent successful HLA‐matched sibling HCT. A homozygous *FANCA* frameshift variant in exon 1 (c.37dupC, p.Gln13Profs*24, NC_000016.9:g.89882986dupG) was identified by exome sequencing. One unaffected sibling was wild‐type and the other was a carrier, but parental DNA was not available for analysis.

Individual 9‐FA presented with neutropenia which progressed to severe BMF and was diagnosed with FA at 10 years of age. Exome sequencing identified and targeted whole gene sequencing validated a large homozygous deletion of exons 7–14 (NC_000016.9:g.89856782_89874222del). Her unaffected sister was a heterozygous carrier. This specific deletion has not been previously reported, but similar large deletions in *FANCA* have been reported (Ameziane et al., 2012).

Affected brothers 17_01‐FA and 17_02‐FA both presented with abnormal thumbs at birth (Figure 2b). Small ear canals were also noted in 17_01‐FA (Figure 2c). At the ages of 6 and 10 years, respectively, they presented with severe BMF and a non‐specific immunodeficiency leading to an FA diagnosis. Biallelic variants in *FANCA* were identified in both siblings (c.2749C>T, p.Arg917*, NC_000016.9:g.89831327G>A, and NC_000016.9:g.89847600‐89853759del). Exome sequencing also revealed a maternally inherited nonsense variant in exon 28 which has been previously identified in an Indian patient with FA and other populations (rs1060501880, ClinVar:408188; Kimble et al., 2018; Solanki et al., 2016). A large deletion of exons 15–17 (NC_000016.9:g.89847600‐89853759del) was detected by targeted PacBio^®^ long‐range sequencing in both affected siblings and has been previously reported in other patients with FA (Gille et al., 2012). This deletion was not detected in DNA from father's peripheral blood, but relatedness analyses confirmed paternity with large regions of homozygosity being consistent with offspring from a consanguineous relationship between third‐degree relatives. Additionally, analyses of single nucleotide polymorphisms (SNP) in the *FANCA* locus provided evidence for a possible genotypic reversion in the paternal hematopoietic stem cells or paternal inheritance as a result of gonadal mosaicism. Both such occurrences have been previously reported in patients with FA (Fargo et al., 2014; Gregory et al., 2001; Gross et al., 2002; Krausz et al., 2019).

Neutropenia developed at 8 years of age and progressed over the next year to severe BMF in individual 19‐FA. A homozygous *FANCA* missense variant in exon 41 (c.4070C>A, p.Ala1357Asp, NC_000016.9:g.89805638G>T) was identified by exome sequencing. Her unaffected brother is a heterozygous carrier, but parental DNA was not available. *FANCA* p Ala1357Asp is not present in gnomAD and is predicted deleterious by *in silico* tools (MetaSVM score = 0.915, REVEL = 0.702, CADD phred = 24.1).

Individual 20‐FA presented with moderate aplastic anemia that progressed to severe BMF by 5 years of age. We identified a homozygous missense variant in *FANCA* (c.1541C>A, p.Ala514Asp, NC_000016.9:g.89849440G>T, rs1432656621). His unaffected sibling is a heterozygous carrier. Although not previously reported, this missense variant is rare in gnomAD at a MAF of 0.0003977% and is predicted deleterious by *in silico* tools (MetaSVM score = 0.876, REVEL = 0.851, CADD phred = 27.1).

### 3.3 *FANCC* homozygous variant and phenotypes

Two unrelated probands, 5‐FA and 8‐FA, were homozygous for the same *FANCC* variant (c.1642C>T, p.Arg548*, NC_000009.11:g.97864024G>A). Relatedness analyses determined that these probands were from distinct families. *FANCC* p.Arg548* (rs104886457, ClinVar:12047) has been previously reported in two FA patients from Pakistan (Aftab et al., 2017).

The clinical features of 5‐FA included moderate aplastic anemia progressing to severe BMF at age11 years. He also had short stature and abnormal left leg growth. He had two brothers and one sister who died due to similar complications but without a diagnosis. His two surviving unaffected siblings and parents are all heterozygous carriers.

Individual 8‐FA presented with moderate aplastic anemia that also progressed to severe BMF by 4 years of age. Skin hyperpigmentation, bone deformities including the absence of metacarpals, thumbs, and radii, and a high arched palate were also reported. He died at the age of 4 years due to a brain hemorrhage before HLA‐matched sibling HCT could be performed. One unaffected sibling is a carrier, but parental DNA was not available.

### 3.4 *FANCF* homozygous variant and phenotype

Individual 10‐FA was homozygous for nonsense variant in *FANCF* (c.785 T>G, p.Leu262*, NC_000011.9:g.22646572A>C, rs368067979). He was diagnosed with FA at 6 years of age when aplastic anemia progressed to severe BMF. He also had polydactyly and died from a hemorrhagic stroke shortly after his FA diagnosis. Parental DNA was not available and the sibling available for testing was not a carrier.

### 3.5 *FANCG* variants and phenotypes

Individual 18‐FA had an extra digit, a small right hand, short stature, and ectopic kidneys. He was diagnosed with FA at 5 years of age and underwent successful HCT from his HLA‐matched sibling for severe BMF. A homozygous nonsense variant in exon 6 of *FANCG* was identified by WES (c.710C>G, p.Ser237*, NC_000009.11:g.35077035G>C). His sibling is wild‐type at this locus. The only parent who was available for testing was heterozygous for this loss of function variant.

Proband 21_01‐FA and his brother 21_02‐FA were diagnosed with FA at the ages of 8 and 5 years, respectively. 21_01‐FA had pancytopenia that progressed rapidly following his diagnosis with FA and he died due to a brain hemorrhage. Currently, 21_02‐FA does not have cytopenias. The bone marrow of both FA‐affected brothers was reported to have numerous rosettes with central eosinophilic material surrounded by small cells seen in a background of fibrosis. The affected brothers have a homozygous frameshift variant in exon 11 of *FANCG* (c.1471_1473delAAAinsG, p.Lys491Glyfs*9, NC_000009.11:g.35075283_35075285delTTTinsC, rs1018027137). One of their unaffected siblings was heterozygous for this variant. This variant has been previously reported in a heterozygous patient with FA (Gille et al., 2012).

### 3.6 *FANCL* variants and phenotypes

Unrelated probands 6‐FA and 7‐FA were both homozygous for a recently identified *FANCL* South Asian founder variant. Donovan *et al*. established this single nucleotide variation (NC_000002.11:g.58387243C>T) induces aberrant mRNA splicing to skip exon 13 (c.1021_1092del, p.Trp341_Lys364del, rs577063114), resulting in a 24 amino acid deletion from the RING domain of FANCL (Donovan et al., 2019). The gnomAD MAF of this variant is 0.001994% in all populations and 0.01634% in South Asian populations. A pairwise comparison between cases 6‐FA and 7‐FA was performed to assess potential relationships. A genotype comparison on approximately 7300 common SNPs between the probands and their siblings showed no indication of relatedness between families 6‐FA and 7‐FA. Parental sequencing data was not available.

Individual 6‐FA had an extra thumb and areas of skin hyperpigmentation. He presented with aplastic anemia progressing to severe BMF at the age of 9 years. Although he was treated with androgens while awaiting an HLA sibling matched HCT, he died at 10 years of age due to an unspecified hemorrhage. Individual 7‐FA was diagnosed with FA after presenting with severe BMF at 7 years of age. He died shortly after his diagnosis at the age of 8 years due to an unreported cause.

### 3.7 Unsolved probands

Rare heterozygous variants in the 22 FA pathway genes were not identified in individual 12‐FA, who was diagnosed with FA by chromosome breakage at the age of 11 years after presenting with BMF. He underwent a successful HLA‐matched sibling HCT and had no reported dysmorphology. Family member DNA was not available to aid in further study.

Variants of potential interest but of uncertain significance were identified in individuals 14‐FA and 16‐FA and are reported in Table S2. FA was diagnosed in proband 14‐FA at 12 year of age and he died 1 year after diagnosis due to an unreported cause. He was a heterozygous carrier for a variant of uncertain significance (VUS) c.583A>G, p.Ile195Val in *FANCN* and a likely benign *FANCO* variant c.‐48C>A. Bilateral thumb malformations were noted at birth in proband 16‐FA who was diagnosed with aplastic anemia at age 9 and underwent a successful HLA‐matched sibling HCT. Heterozygous VUS were present in *FANCA*, *FANCD2*, *FANCI*, and *FANCP*. The *FANCP* variant (c.2209C>T, p.Arg737Cys, NC_000016.9:g.3642818G>A, rs140706384) may be deleterious as it has a REVEL score of 0.449 and a CADD score of 26.2, but the MetaSVM score was predicted as tolerated; additional functional studies are required to determine potential pathogenicity. There were no other deleterious variants or large CNV events detected in *FANCP*.

### 3.8 Variants reported in individuals with FA from South Asia and the Middle East

**FIGURE 3:**
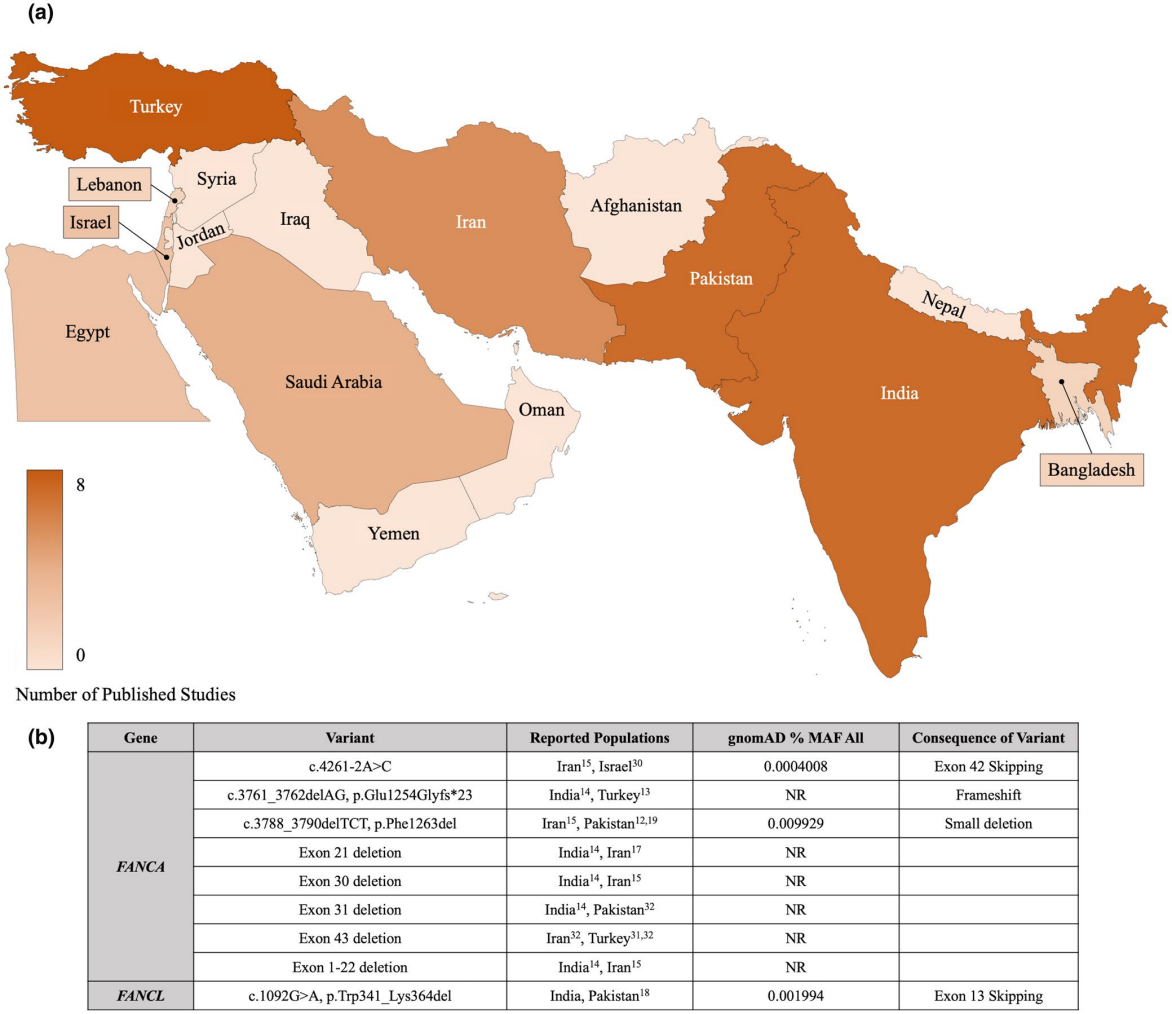
(a) A heat map showing the distribution of studies published from South Asia and the Middle East on the genetic etiology of FA. *This heat map was generated by the primary author using a tool available in Microsoft PowerPoint* *(v16.40)*. (b) The variants reported to cause FA in multiple populations in South Asia and the Middle East. MAF, minor allele frequency; NR, not reported

We identified 29 published reports with data on gene variants in individuals with FA from South Asia and the Middle East. Reports from Turkey, India, and Pakistan were the most common, followed by Iran and Saudi Arabia (Figure 3a). Tables S3–S5 list the specific large deletions, single nucleotide variants, and small insertions and deletions, respectively, along with the relevant references. Ten of the 29 publications reported on targeted sequencing of only the *FANCA* gene. The other publications performed a combination of hotspot variant analysis, targeted gene sequencing, Sanger sequencing, RNA sequencing, other next generation sequencing methods, and multiplex ligation‐dependent probe amplification analysis. Variants reported in more than one population are noted in Figure 3b.

The majority of reported variants occurred in *FANCA* *(78%)* and were private to their respective populations. Variants reported in *FANCG*, *FANCC*, and *FANCE* accounted for 7.8%, 2.8%, and 2.8% of the total variants reported. The only large deletions reported were in *FANCA*, similar to our findings and consistent with others. Donovan et al recently identified a *FANCL* founder variant in South Asian populations (c.1092G>A, p.Trp341_Lys364del; Donovan et al., 2019). We identified this same founder variant in two, unrelated families from Pakistan in our cohort.

## 4 DISCUSSION

The identification of the genetic causes of rare diseases such as FA is important to verify diagnoses, improve clinical management, allow for appropriate genetic counseling, and understand their underlying pathobiology. The genetic cause of FA was identified in 16 patients from 14 families in this study; three families remain molecularly undiagnosed. *FANCA* was the most commonly affected gene with pathogenic *FANCA* variants present in 50% of the families. The other pathogenic variants were present in *FANCC*, *FANCG*, or *FANCL* (2 families each), and *FANCF* (1 family). Only 6 of the 14 variants identified in our study were present in any gnomAD population and 3 of these were solely in South Asian populations (Table 2).

Homozygosity for pathogenic variants was present in 12 of the 14 families. Only two families had compound heterozygous inheritance (both in *FANCA*). Two families from the Central Punjab region of Pakistan were homozygous for the *FANCL* founder variant (Donovan et al., 2019). We were unable to evaluate consanguinity in 14 of the families in this study due to lack of parental DNA samples. However, the presence of homozygous pathogenic variants in our data is consistent with prior studies reporting an approximately 70% rate of consanguineous marriages in Pakistan (Hussain & Bittles, 1998; Mobarak et al., 2019; National Institute of Population, 2013). Nine of the 14 pathogenic variants identified in this study have been previously reported (Table 2; Aftab et al., 2017; Castella et al., 2011; Donovan et al., 2019; Esmail Nia et al., 2016; Gille et al., 2012; Solanki et al., 2016).

While our study is limited to individuals with FA and BMF severe enough to warrant HCT evaluation, it is one of a very few studies seeking to understand the type and frequencies of FA‐associated germline variants in the Pakistani population. We expect that the individuals included in this study represent a small subset of FA cases in Pakistan. It is possible that in the absence of overt dysmorphology, the diagnosis of FA may be delayed or missed because chronic malnutrition could confound diagnoses such as anemia and even overt BMF (National Institute of Population, 2013).

The majority of studies published, to date, on the genetics of FA in South Asia and the Middle East focused primarily on targeted sequencing of *FANCA*, with *FANCC*, *FANCG*, and *FANCE* being evaluated but less frequently. *FANCA*, *FANCC*, and *FANCG* account for upwards of 85% of FA cases in individuals of northern European ancestry but the relatively small number of studies from other ethnic groups limits our understanding of the population genetics of FA‐associated genes and the full scope of FA around the world. A limited number of FA genotype‐phenotype studies suggest there may be phenotypic differences based on the associated genetic etiology (Fiesco‐Roa et al., 2019). The presence of founder mutations in *FANCA*, *FANCG*, and *FANCL* warrants additional study in the context of biology and clinical manifestations.

Large genotype‐phenotype studies of patients with FA around the world as well as population‐based sequencing efforts are required to better understand the genetic variation of the FA‐associated DNA repair genes in diverse populations in order to uncover disease etiology, improve diagnostics and patient management, and to provide appropriate genetic counseling.

## AUTHORS’ CONTRIBUTIONS

Data acquisition: NS, SM, PA, SS, SR; data analysis and interpretation: AST, LJM, KJ, MY, FXD, SCC, BPA, SAS, SR; drafted manuscript: AST, SAS; and all authors approved of the final manuscript.

## Supporting information

Table S1

Table S2

Table S3

Table S4

Table S5

## Data Availability

The deidentified genomic data generated in this study are available upon reasonable request from Dr. Sadia Rehman (sadiawasiq@gmail.com, Institute of Biomedical and Genetic Engineering, Islamabad, Pakistan) through collaboration agreements.
