# Supplementary material for: The causes of Fanconi anemia in South Asia and the Middle East: A case series and review of the literature
Source: Mol Genet Genomic Med. 2021 May 7;9(7):e1693. doi: 10.1002/mgg3.1693 (PMC8372062; doi:10.1002/mgg3.1693)
Supplement: Supplementary file 1 — Table S1 [file MGG3-9-e1693-s004.docx]

**SUPPORTING INFORMATION**

**SUPPLEMENTARY TABLE 1** Characteristics of study participants.

| **Family ID** | **Relation to Proband** | **Gender** | **Affected Status** |
| --- | --- | --- | --- |
| 1FA | Proband | M | A |
|  | Sibling | M | U |
|  | Father | M | U |
|  | Mother | F | U |
| 3FA | Proband | M | A |
|  | Sibling | M | U |
|  | Sibling | M | U |
|  | Father | M | U |
|  | Mother | F | U |
| 4FA | Proband | M | A |
|  | Sibling | M | U |
|  | Sibling | M | U |
| 5FA | Proband | M | A |
|  | Sibling | M | U |
|  | Sibling | M | U |
|  | Mother | F | U |
|  | Father | M | U |
| 6FA | Proband | M | A |
|  | Sibling | M | U |
| 7FA | Proband | M | A |
|  | Sibling | M | U |
| 8FA | Proband | M | A |
|  | Sibling | F | U |
|  | Sibling | F | U |
| 9FA | Proband | F | A |
|  | Sibling | F | U |
|  | Sibling | F | U |
| 10FA | Proband | M | A |
|  | Sibling | F | U |
| 12FA | Proband | M | A |
|  | Sibling | M | U |
| 14FA | Proband | M | A |
|  | Sibling | F | U |
| 16FA | Proband | F | A |
|  | Sibling | M | U |
|  | Sibling | F | U |
|  | Sibling | F | U |
| 17FA | Proband | M | A |
|  | Sibling | M | A |
|  | Sibling | F | U |
|  | Father | M | U |
|  | Mother | F | U |
| 18FA | Proband | M | A |
|  | Sibling | F | U |
|  | Mother | F | U |
| 19FA | Proband | F | A |
|  | Sibling | M | U |
| 20FA | Proband | M | A |
|  | Sibling | M | U |
| 21FA | Proband | F | A |
|  | Sibling | M | A |
|  | Sibling | M | U |

*Abbreviations*: M, Male; F, Female; A, Affected; U, Unaffected.
