# Supplementary material for: The causes of Fanconi anemia in South Asia and the Middle East: A case series and review of the literature
Source: Mol Genet Genomic Med. 2021 May 7;9(7):e1693. doi: 10.1002/mgg3.1693 (PMC8372062; doi:10.1002/mgg3.1693)
Supplement: Supplementary file 2 — Table S2 [file MGG3-9-e1693-s001.docx]

**SUPPORTING INFORMATION**

**SUPPLEMENTARY TABLE 2.** Rare heterozygous variants identified in the unsolved patients.

| **Patient ID** | **Gene** | **Variant** | **ClinVar** | **gnomAD % MAF – General Population** | **gnomAD % MAF – South Asian** | **REVEL** | **MetaSVM** | **CADD** |
| --- | --- | --- | --- | --- | --- | --- | --- | --- |
| 14FA | *FANCN* | c.583A>G, p.Ile195Val | VUS | 0.007955 | 0.06534 | 0.01 | -0.987 | 0.113 |
|  | *FANCO* † | c.-48C>A | Benign | 0.02065 | 0.07198 |  |  |  |
| 16FA | *FANCA* | c.1360-172G>A | NR |  |  |  |  |  |
|  | *FANCD2* | c.27A>C, p.Lys9Asn | NR |  |  | 0.068 | -1.025 | 8.054 |
|  | *FANCI* | c.1583+6T>C | NR |  |  |  |  |  |
|  | *FANCP* | c.2209C>T, p.Arg737Cys | NR | 0.000799 | 0.006535 | 0.449 | -0.07 | 26.2 |

† Indicates the variant was reported in other populations, but the minor allele frequency (MAF) was second highest in those of South Asian descent. Blank cells indicate the variant was not present in gnomAD.

*Abbreviations*: VUS, Variant of uncertain significance; NR, Not reported.
