# Supplementary material for: The causes of Fanconi anemia in South Asia and the Middle East: A case series and review of the literature
Source: Mol Genet Genomic Med. 2021 May 7;9(7):e1693. doi: 10.1002/mgg3.1693 (PMC8372062; doi:10.1002/mgg3.1693)
Supplement: Supplementary file 3 — Table S3 [file MGG3-9-e1693-s003.docx]

**SUPPORTING INFORMATION**

**SUPPLEMENTARY TABLE 3** All large deletions reported in patients with FA in South Asia and the Middle East.

| **Population** | **Gene** | **Chromosome** | **Large Deletions** | **Reference** |
| --- | --- | --- | --- | --- |
| Egypt | *FANCA* | chr16 | Exon 31-32 | (Wijker et al., 1999) |
| India | *FANCA* | chr16 | Exon 8-27 | (Shukla, Rao, Ghosh, & Vundinti, 2013) |
|  |  |  | Exon 7-31 | (Wijker et al., 1999) |
|  |  |  | Exon 11 | (Solanki et al., 2016) |
|  |  |  | Exon 21 |  |
|  |  |  | Exon 1-8 |  |
|  |  |  | Exon 8-27 |  |
|  |  |  | Exon 30 |  |
|  |  |  | Exon 15-29 |  |
|  |  |  | Exon 1-22 |  |
|  |  |  | Exon 31 |  |
|  |  |  | Exon 4-7 |  |
|  |  |  | Exon 6 |  |
| Iran | *FANCA* | chr16 | Exon 8-43 | (Donovan et al., 2019) |
|  |  |  | Exon 1-30 | (Wijker et al., 1999) |
|  |  |  | Exon 43 |  |
|  |  |  | Exon 1-22 | (Esmail Nia, Fadaee, Royer, Najmabadi, & Akbari, 2016) |
|  |  |  | Exon 30 |  |
|  |  |  | Exon 1-44 |  |
|  |  |  | Exon 1-26 |  |
|  |  |  | Exon 18-20 |  |
|  |  |  | Exon 7 |  |
|  |  |  | Exon 20-22 |  |
|  |  |  | Exon 20-28 |  |
|  |  |  | Exon 16-22 |  |
|  |  |  | Exon 21 | (Gille et al., 2012) |
| Israel | *FANCA* | chr16 | Exon 6-31 | (Tamary et al., 2004) |
| Pakistan | *FANCA* | chr16 | Exon 31 | (Wijker et al., 1999) |
| Turkey | *FANCA* | chr16 | Exon 43 | (Koc, Pronk, Alikasifoglu, Joenje, & Altay, 1999; Wijker et al., 1999) |

**REFERENCES**

Donovan, F. X., Solanki, A., Mori, M., Chavan, N., George, M., C, S. K., . . . Vundinti, B. R. (2019). A founder variant in the South Asian population leads to a high prevalence of FANCL Fanconi anemia cases in India. *Hum Mutat*. doi:10.1002/humu.23914

Esmail Nia, G., Fadaee, M., Royer, R., Najmabadi, H., & Akbari, M. R. (2016). Profiling Fanconi Anemia Gene Mutations among Iranian Patients. *Arch Iran Med, 19*(4), 236-240. doi:0161904/AIM.003

Gille, J. J., Floor, K., Kerkhoven, L., Ameziane, N., Joenje, H., & de Winter, J. P. (2012). Diagnosis of Fanconi Anemia: Mutation Analysis by Multiplex Ligation-Dependent Probe Amplification and PCR-Based Sanger Sequencing. *Anemia, 2012*, 603253. doi:10.1155/2012/603253

Koc, A., Pronk, J. C., Alikasifoglu, M., Joenje, H., & Altay, C. (1999). Variable pathogenicity of exon 43del (FAA) in four Fanconi anaemia patients within a consanguineous family. *Br J Haematol, 104*(1), 127-130. doi:10.1046/j.1365-2141.1999.01156.x

Shukla, P., Rao, A., Ghosh, K., & Vundinti, B. R. (2013). Identification of a novel large intragenic deletion in a family with Fanconi anemia: first molecular report from India and review of literature. *Gene, 518*(2), 470-475. doi:10.1016/j.gene.2013.01.016

Solanki, A., Mohanty, P., Shukla, P., Rao, A., Ghosh, K., & Vundinti, B. R. (2016). FANCA Gene Mutations with 8 Novel Molecular Changes in Indian Fanconi Anemia Patients. *PLoS One, 11*(1), e0147016. doi:10.1371/journal.pone.0147016

Tamary, H., Dgany, O., Toledano, H., Shalev, Z., Krasnov, T., Shalmon, L., . . . Yaniv, I. (2004). Molecular characterization of three novel Fanconi anemia mutations in Israeli Arabs. *Eur J Haematol, 72*(5), 330-335. doi:10.1111/j.1600-0609.2004.00240.x

Wijker, M., Morgan, N. V., Herterich, S., van Berkel, C. G., Tipping, A. J., Gross, H. J., . . . et al. (1999). Heterogeneous spectrum of mutations in the Fanconi anaemia group A gene. *Eur J Hum Genet, 7*(1), 52-59. doi:10.1038/sj.ejhg.5200248
