# Supplementary material for: The causes of Fanconi anemia in South Asia and the Middle East: A case series and review of the literature
Source: Mol Genet Genomic Med. 2021 May 7;9(7):e1693. doi: 10.1002/mgg3.1693 (PMC8372062; doi:10.1002/mgg3.1693)
Supplement: Supplementary file 4 — Table S4 [file MGG3-9-e1693-s005.docx]

**SUPPORTING INFORMATION**

**SUPPLEMENTARY TABLE 4** All single nucleotide variants reported in patients with FA in South Asia and the Middle East. Genomic positions for the variants listed in red could not be identified based on their original report.

| Population | Gene | Chromosome | Position (hg19) | Variant | | rsID | Reference |
| --- | --- | --- | --- | --- | --- | --- | --- |
| Bangladesh | FANCE | chr6 | 35423696 | c.421C>T | p.Arg141* | rs121434506 | (de Winter et al., 2000) |
| Egypt | FANCA | chr16 | 89871683 | c.709+5G>T |  | rs759877008 | (Wijker et al., 1999) |
|  |  |  | No variants to report in Exons 27, 34, or 43 | | |  | (Salem et al., 2014) |
| India | FANCA | chr16 | 89833576 | c.2574C>G | p.Ser858Arg | rs17233141 | (Tamary et al., 2000) |
|  |  |  | 89862427 | c.894-1G>A |  |  | (Wijker et al., 1999) |
|  |  |  | 89816214 | c.3163C>T | p.Arg1055Trp | rs753063086 |  |
|  |  |  | 89805672 | c.4036G>A | p.Ala1346Thr | rs17227396 | (Solanki et al., 2016) |
|  |  |  | 89809296 | c.3678C>G | p.Ser1226* |  |  |
|  |  |  | 89805904 | c.3992T>C | p.Leu1331Pro |  |  |
|  |  |  | 89857897 | c.1273G>C | p.Asp425His | rs1555561398 |  |
|  |  |  | 89833576 | c.2574C>G | p.Ser858Arg | rs17233141 |  |
|  |  |  | 89857867 | c.1303C>T | p.Arg435Cys | rs148473140 |  |
|  |  |  | 89831446 | c.2630C>G | p.Ser877* |  |  |
|  |  |  | 89882311 | c.163C>T | p.Gln55* | rs1555580427 |  |
|  |  |  | 89816189 | c.3189G>A | p.Trp1063* | rs1166286386 |  |
|  |  |  | 89809294 | c.3679G>C | p.Ala1227Pro |  |  |
|  |  |  | 89831327 | c.2749C>T | p.Arg917* | rs1060501880 |  |
|  |  |  | 89828358 | c.2851C>T | p.Arg951Trp | rs755546887 |  |
|  |  |  | 89837012 | c.2182C>T | p.Gln728* |  |  |
|  |  |  | 89809296 | c.3678C>G | p.Ser1226* |  | (Vundinti, 2014) |
|  |  |  | 89805903 | c.3993G>A | p.Leu1331Pro | rs1158871974 |  |
|  |  |  |  | c.1274C>G | p.Glu425His |  |  |
|  |  |  | 89831446 | c.2630C>G | p.Ser877* |  |  |
|  |  |  | 89866043 | c.796A>G | p.Thr266Ala | rs7190823 |  |
|  |  |  | 89849480 | c.1501G>A | p.Gly501Ser | rs2239359 |  |
|  |  |  | 89836323 | c.2426G>A | p.Gly809Asp | rs7195066 |  |
|  | FANCG | chr9 | 35075954 | c.1143+5G>C | p.Arg359Serfs*22 | rs778328620 | (Solanki et al., 2017) |
|  | FANCI | chr15 | 89803950 | c.164C>T | p.Pro55Leu | rs62020347 | (Dorsman et al., 2007) |
|  |  |  | 89858550 | c.3854G>A | p.Arg1285Gln | rs121918163 |  |
|  | FANCL | chr2 | 58387258-58388671 | c.1021_1092del | p.Trp341_Lys364del |  | (Donovan et al., 2019) |
| Iran | FANCA | chr16 | 89805563 | c.4145G>A | p.Arg1382Lys | rs367970303 | (Moghadam, Mahjoubi, Reisi, & Vosough, 2016) |
|  |  |  | 89805662 | c.4046G>C | p.Arg1349Thr |  |  |
|  |  |  | 89805914 | c.3982A>G | p.Thr1328Ala | rs9282681 |  |
|  |  |  | 89815152 | c.3263C>T | p.Ser1088Phe | rs17233497 | (Wijker et al., 1999) |
|  |  |  | 89825051 | c.2915G>T | p.Gly972Val |  | (Esmail Nia, Fadaee, Royer, Najmabadi, & Akbari, 2016) |
|  |  |  | 89805890 | c.4006T>G | p.Tyr1336Asp |  |  |
|  |  |  | 89881023 | c.190-2A>G |  |  |  |
|  |  |  | 89815066 | c.3348+1G>T |  | rs751266148 |  |
|  |  |  | 89815066 | c.3348+1G>A | Skipping of Exon 33 | rs751266148 |  |
|  |  |  | 89805118 | c.4261-2A>C | Skipping of Exon 42 | rs915983602 |  |
|  |  |  | 89815129 | c.3286C>T | p.Gln1096* | rs775799529 |  |
|  | FANCD2 | chr3 | 10108951 | c.2444G>A | p.Arg815Gln | rs766567785 |  |
|  | FANCG | chr9 | 35079240 | c.85-2A>T |  | rs759590778 |  |
|  |  |  | 35078137 | c.510+1G>T |  |  |  |
|  |  |  | 35078601 | c.307+1G>C |  | rs200479612 | (Gille et al., 2012) |
|  | FANCL | chr2 | 58453921 | c.217-2A>G |  | rs755417959 | (Esmail Nia et al., 2016) |
| Israel | FANCA | chr16 | 89805118 | c.4261-2A>C | Skipping of Exon 42 | rs915983602 | (Tamary et al., 2004) |
|  |  |  |  | c.719A>G | p.Val229Ile |  |  |
|  | FANCG | chr9 | 35078135 | c.510+3A>G |  |  |  |
| Lebanon | FANCA | chr16 | 89838130 | c.2107C>T | p.Gln703* | rs1555548512 | (Wijker et al., 1999) |
| Pakistan | FANCA | chr16 | 89805378 | c.4172A>G | p.Asn1391Ser | rs1385967375 | (Shahid et al., 2019) |
|  |  |  | 89805334 | c.4216T>G | p.Leu1406Val |  |  |
|  |  |  | 89805324 | c.4226G>C | p.Arg1409Pro |  |  |
|  |  |  | 89805311 | c.4239G>T | p.Lys1413Asn |  |  |
|  |  |  | 89805298 | c.4252G>A | p.Val1418Met | rs145148206 |  |
|  |  |  | 89805086 | c.4291G>C | p.Glu1431Gln | rs1567591125 |  |
|  |  |  | 89805066 | c.4311G>T | p.Gln1437His |  |  |
|  |  |  | 89805061 | c.4316G>A | p.Arg1439Lys | rs587778322 |  |
|  |  |  | 89805056 | c.4321C>A | p.Gln1441Lys |  |  |
|  |  |  | 89805049 | c.4328C>G | p.Ala1443Gly |  |  |
|  |  |  | 89805029 | c.4348C>G | p.Gln1450Glu |  |  |
|  |  |  | 89809319 | c.3654A>G | p.Pro1218= | rs1800358 |  |
|  |  |  | 89807233 | c.3807G>C | p.Leu1269= | rs11649210 |  |
|  |  |  | 89805102 | c.4275T>A | p.Arg1425= |  |  |
|  |  |  | 89805114 | c.4263G>A | p.Leu1421= |  |  |
|  | FANCC | chr9 | 97934315 | c.456+4A>T |  | rs104886456 | (Aftab et al., 2017) |
|  |  |  | 97864024 | c.1642C>T | p.Arg548* | rs104886457 |  |
|  | FANCG | chr9 | 35078335 | c.313G>T | p.Glu105* | rs121434425 | (Aymun et al., 2017) |
|  | FANCL | chr2 | 58387258-58388671 | c.1021_1092del | p.Trp341_Lys364del |  | (Donovan et al., 2019) |
|  | FANCO/RAD51C | chr17 | 56787287 | c.773G>A | p.Arg258His | rs267606997 | (Vaz et al., 2010) |
| Saudi Arabia | FANCA | chr16 | 89883000 | c.24C>G | p.Asn8Lys | rs76275444 | (Levran et al., 1997) |
|  | FANCC | chr9 | 97869406 | c.1475T>C | p.Leu492Pro |  | (Ghazwani et al., 2016) |
|  | FANCD1/BRCA2 | chr13 | 32921033 | c.7007G>A | p.Arg2336His | rs28897743 |  |
|  | FANCJ/BRIP1 | chr17 | 59793412 | c.2932C>T | p.Arg798* | rs137852986 |  |
|  | FANCU/XRCC2 | chr7 | 152345927 | c.643C>T | p.Arg215* | rs143153871 | (Shamseldin, Elfaki, & Alkuraya, 2012) |
| Turkey | FANCD2 | chr3 | 10106024 | c.1948-16T>G | p.Glu650* | rs17233141 | (Kalb et al., 2007) |
|  | FANCE | chr6 | First report of FANCE; Specific variant not reported | | |  | (Wegner, Henrichs, Joenje, & Schroeder-Kurth, 1996) |
|  |  |  | 35423630 | c.355C>T | p.Gln119* | rs121434505 | (de Winter et al., 2000) |
|  |  |  | 35427100 | c.1114-8G>A | p.Ile372Leufs* | rs878854342 |  |
|  | FANCI | chr15 | 89790880 | c.2T>C |  | rs121434505 | (Dorsman et al., 2007) |

**REFERENCES**

Aftab, I., Iram, S., Khaliq, S., Israr, M., Ali, N., Jahan, S., . . . Mohsin, S. (2017). Analysis of FANCC gene mutations (IVS4+4A>T, del322G, and R548X)in patients with Fanconi anemia in Pakistan. Turk J Med Sci, 47(2), 391-398. doi:10.3906/sag-1506-53

Aymun, U., Iram, S., Aftab, I., Khaliq, S., Nadir, A., Nisar, A., & Mohsin, S. (2017). Screening for mutations in two exons of FANCG gene in Pakistani population. Biomed Pap Med Fac Univ Palacky Olomouc Czech Repub, 161(2), 158-163. doi:10.5507/bp.2017.030

de Winter, J. P., Leveille, F., van Berkel, C. G., Rooimans, M. A., van Der Weel, L., Steltenpool, J., . . . Joenje, H. (2000). Isolation of a cDNA representing the Fanconi anemia complementation group E gene. Am J Hum Genet, 67(5), 1306-1308. doi:10.1016/S0002-9297(07)62959-0

Donovan, F. X., Solanki, A., Mori, M., Chavan, N., George, M., C, S. K., . . . Vundinti, B. R. (2019). A founder variant in the South Asian population leads to a high prevalence of FANCL Fanconi anemia cases in India. Hum Mutat. doi:10.1002/humu.23914

Dorsman, J. C., Levitus, M., Rockx, D., Rooimans, M. A., Oostra, A. B., Haitjema, A., . . . Joenje, H. (2007). Identification of the Fanconi anemia complementation group I gene, FANCI. Cell Oncol, 29(3), 211-218. doi:10.1155/2007/151968

Esmail Nia, G., Fadaee, M., Royer, R., Najmabadi, H., & Akbari, M. R. (2016). Profiling Fanconi Anemia Gene Mutations among Iranian Patients. Arch Iran Med, 19(4), 236-240. doi:0161904/AIM.003

Ghazwani, Y., AlBalwi, M., Al-Abdulkareem, I., Al-Dress, M., Alharbi, T., Alsudairy, R., . . . Alsultan, A. (2016). Clinical characteristics and genetic subtypes of Fanconi anemia in Saudi patients. Cancer Genet, 209(4), 171-176. doi:10.1016/j.cancergen.2016.02.003

Gille, J. J., Floor, K., Kerkhoven, L., Ameziane, N., Joenje, H., & de Winter, J. P. (2012). Diagnosis of Fanconi Anemia: Mutation Analysis by Multiplex Ligation-Dependent Probe Amplification and PCR-Based Sanger Sequencing. Anemia, 2012, 603253. doi:10.1155/2012/603253

Kalb, R., Neveling, K., Hoehn, H., Schneider, H., Linka, Y., Batish, S. D., . . . Schindler, D. (2007). Hypomorphic mutations in the gene encoding a key Fanconi anemia protein, FANCD2, sustain a significant group of FA-D2 patients with severe phenotype. Am J Hum Genet, 80(5), 895-910. doi:10.1086/517616

Levran, O., Erlich, T., Magdalena, N., Gregory, J. J., Batish, S. D., Verlander, P. C., & Auerbach, A. D. (1997). Sequence variation in the Fanconi anemia gene FAA. Proc Natl Acad Sci U S A, 94(24), 13051-13056. doi:10.1073/pnas.94.24.13051

Moghadam, A. A., Mahjoubi, F., Reisi, N., & Vosough, P. (2016). Investigation of FANCA gene in Fanconi anaemia patients in Iran. Indian J Med Res, 143(2), 184-196. doi:10.4103/0971-5916.180206

Salem, A. M., El-Bassyouni, H. T., El-Kamah, G. Y., Zarouk, W. A., Eid, M. M., Mosaad, R. M., . . . Temtamy, S. A. (2014). Screening for common mutations in four FANCA gene exons in Egyptian Fanconi anemia patients. Middle East Journal of Medical Genetics, 3(1), 24-30. doi:10.1097/01.MXE.0000438179.47299.3c

Shahid, M., Firasat, S., Satti, H. S., Satti, T. M., Ghafoor, T., Sharif, I., & Afshan, K. (2019). Screening of the FANCA gene mutational hotspots in the Pakistani fanconi anemia patients revealed 19 sequence variations. Congenit Anom (Kyoto). doi:10.1111/cga.12331

Shamseldin, H. E., Elfaki, M., & Alkuraya, F. S. (2012). Exome sequencing reveals a novel Fanconi group defined by XRCC2 mutation. J Med Genet, 49(3), 184-186. doi:10.1136/jmedgenet-2011-100585

Solanki, A., Kumar Selvaa, C., Sheth, F., Radhakrishnan, N., Kalra, M., & Vundinti, B. R. (2017). Characterization of two novel FANCG mutations in Indian Fanconi anemia patients. Leuk Res, 53, 50-56. doi:10.1016/j.leukres.2016.11.013

Solanki, A., Mohanty, P., Shukla, P., Rao, A., Ghosh, K., & Vundinti, B. R. (2016). FANCA Gene Mutations with 8 Novel Molecular Changes in Indian Fanconi Anemia Patients. PLoS One, 11(1), e0147016. doi:10.1371/journal.pone.0147016

Tamary, H., Bar-Yam, R., Shalmon, L., Rachavi, G., Krostichevsky, M., Elhasid, R., . . . Zaizov, R. (2000). Fanconi anaemia group A (FANCA) mutations in Israeli non-Ashkenazi Jewish patients. Br J Haematol, 111(1), 338-343. doi:10.1046/j.1365-2141.2000.02323.x

Tamary, H., Dgany, O., Toledano, H., Shalev, Z., Krasnov, T., Shalmon, L., . . . Yaniv, I. (2004). Molecular characterization of three novel Fanconi anemia mutations in Israeli Arabs. Eur J Haematol, 72(5), 330-335. doi:10.1111/j.1600-0609.2004.00240.x

Vaz, F., Hanenberg, H., Schuster, B., Barker, K., Wiek, C., Erven, V., . . . Mathew, C. G. (2010). Mutation of the RAD51C gene in a Fanconi anemia-like disorder. Nat Genet, 42(5), 406-409. doi:10.1038/ng.570

Vundinti, B. R. (2014). Chromosomal instability and molecular mutations in multi spectrum disease of Fanconi anemia. Mol Cytogenet, 7(Suppl 1 Proceedings of the International Conference on Human), I47. doi:10.1186/1755-8166-7-S1-I47

Wegner, R. D., Henrichs, I., Joenje, H., & Schroeder-Kurth, T. (1996). Fanconi anemia complementation group E: clinical and cytogenetic data of the first patient. Clin Genet, 50(6), 479-482. doi:10.1111/j.1399-0004.1996.tb02716.x

Wijker, M., Morgan, N. V., Herterich, S., van Berkel, C. G., Tipping, A. J., Gross, H. J., . . . et al. (1999). Heterogeneous spectrum of mutations in the Fanconi anaemia group A gene. Eur J Hum Genet, 7(1), 52-59. doi:10.1038/sj.ejhg.5200248
