# Supplementary material for: The causes of Fanconi anemia in South Asia and the Middle East: A case series and review of the literature
Source: Mol Genet Genomic Med. 2021 May 7;9(7):e1693. doi: 10.1002/mgg3.1693 (PMC8372062; doi:10.1002/mgg3.1693)
Supplement: Supplementary file 5 — Table S5 [file MGG3-9-e1693-s002.docx]

**SUPPORTING INFORMATION**

**SUPPLEMENTARY TABLE 5** All small insertion/deletion variants reported in patients with FA in South Asia and the Middle East.

| **Population** | **Gene** | **Chromosome** | **Position (hg19)** | **Variant** | | **rsID** | **Reference** |
| --- | --- | --- | --- | --- | --- | --- | --- |
| India | *FANCA* | chr16 | 89809211-89809212 | c.3761_3762delAG | p.Glu1254Glyfs*23 | rs868273545 | (Solanki et al., 2016) |
|  |  |  | 89806407-89806410 | c.3926_3929delCAGA | p.Thr1309Argfs* |  |  |
|  |  |  | 89836249 | c.2500delC | p.Leu384* |  |  |
|  |  |  | 89836252-89836254 | c.2495_2497delTCT | p.Phe832del | rs1310756192 | (Wijker et al., 1999) |
|  | *FANCG* | chr9 | 35076762 | c.883dupG | p.Asp295Glyfs*14 |  | (Solanki et al., 2017) |
|  |  |  | 35075282-35075285 | c.1471_1473delAAAinsG | p.Lys491Glyfs*9 |  |  |
| Iran | *FANCA* | chr16 | 89805659-89805660 | c.4048_4049insT | p.Glu1350Valfs* |  | (Moghadam, Mahjoubi, Reisi, & Vosough, 2016) |
|  |  |  | 89805661-89805662 | c.4046_4047insT | p.Arg1349Serfs* |  |  |
|  |  |  | 89805686 | c.4022dupA | p.Tyr1341* |  |  |
|  |  |  | 89807249-89807251 | c.3791_3793delCCT | p.Ser1264del |  | (Esmail Nia, Fadaee, Royer, Najmabadi, & Akbari, 2016) |
|  |  |  | 89805903 | c.3993delG | p.Pro1332Leufs*31 |  |  |
|  |  |  | 89807255-89805259 | c. 3781_3785delTTCTT | p.Phe1261Leufs*15 |  |  |
|  |  |  | 89805583-89805584 | c.4124_4125delCA | p.Thr1375Serfs*49 | rs776969626 |  |
|  |  |  | 89807250-89807252 | c.3788_3790delTCT | p.Phe1263del | rs397507553 |  |
|  |  |  | 89862330-89862333 | c.987-990delTCAC | p.His330Alafs*4 | rs772359099 |  |
|  |  |  | 89809279 | c.3639delT | p.Phe1232fs | rs1555535527 |  |
|  | *FANCF* | chr11 | 22646823 | c.534delG | p.Lys179Argfs*24 |  | (Zareifar et al., 2019) |
|  | *FANCG* | chr9 | 35077096-35077100 | c.647-2_649delAGGTC |  |  | (Esmail Nia et al., 2016) |
|  |  |  | 35074201 | c.1772delT | p.Leu591Argfs*3 |  |  |
| Israel | *FANCA* | chr16 | 89837021-89837022 | c.2172dup | p.Ser725Valfs*28 | rs1555547955 | (Tamary et al., 2000) |
| Pakistan | *FANCA* | chr16 | 89807250-89807252 | c.3788_3790delTCT | p.Phe1263del | rs397507553 | (Castella et al., 2011) |
|  |  |  | 89809265 | c.3708dup | p.Val1237Serfs*41 |  | (Shahid et al., 2019) |
|  |  |  | 89807250-89807252 | c.3788_3790delTCT | p.Phe1263del | rs397507553 |  |
|  |  |  | 89806482 | c.3854dupA | p.His1286Profs*27 |  |  |
|  |  |  | 89806464 | c.3872dupA | p.Leu1292Profs*21 |  |  |
|  | *FANCC* | chr9 | 98002954 | c.322delA | p.Ser108Glnfs* |  | (Aftab et al., 2017) |
| Saudi Arabia | *FANCG* | chr9 | 35077267-35077273 | c.637_643del | p.Tyr213Lysfs*6 | rs587776640 | (Ghazwani et al., 2016) |
|  | *FANCN/PALB2* | chr16 | 23614916 | c.3425del | p.Leu1142Tyrfs*21 | rs1057517563 |  |
| Turkey | *FANCA* | chr16 | 89809334 | c.3639delT | p.Glu1214Argfs* |  | (Balta, de Winter, Kayserili, Pronk, & Joenje, 2000) |
|  |  |  | 89809211-89809212 | c.3761_3762delAG | p.Glu1254Glyfs*23 | rs868273545 | (Levran et al., 1997) |
|  |  |  | 89849278 | c.1615delG | p.Asp539Thrfs*66 | rs778507965 | (Aslan, Ameziane, & De Winter, 2015) |

**REFERENCES**

Aftab, I., Iram, S., Khaliq, S., Israr, M., Ali, N., Jahan, S., . . . Mohsin, S. (2017). Analysis of FANCC gene mutations (IVS4+4A>T, del322G, and R548X)in patients with Fanconi anemia in Pakistan. *Turk J Med Sci, 47*(2), 391-398. doi:10.3906/sag-1506-53

Aslan, D., Ameziane, N., & De Winter, J. P. (2015). Molecular diagnosis of Fanconi anemia with next-generation sequencing in a case with subtle signs and a negative chromosomal breakage test. *Turk J Pediatr, 57*(3), 282-285. Retrieved from <https://www.ncbi.nlm.nih.gov/pubmed/26701949>

Balta, G., de Winter, J. P., Kayserili, H., Pronk, J. C., & Joenje, H. (2000). Fanconi anemia A due to a novel frameshift mutation in hotspot motifs: lack of FANCA protein. *Hum Mutat, 15*(6), 578. doi:10.1002/1098-1004(200006)15:6<578::AID-HUMU12>3.0.CO;2-Q

Castella, M., Pujol, R., Callen, E., Trujillo, J. P., Casado, J. A., Gille, H., . . . Surralles, J. (2011). Origin, functional role, and clinical impact of Fanconi anemia FANCA mutations. *Blood, 117*(14), 3759-3769. doi:10.1182/blood-2010-08-299917

Esmail Nia, G., Fadaee, M., Royer, R., Najmabadi, H., & Akbari, M. R. (2016). Profiling Fanconi Anemia Gene Mutations among Iranian Patients. *Arch Iran Med, 19*(4), 236-240. doi:0161904/AIM.003

Ghazwani, Y., AlBalwi, M., Al-Abdulkareem, I., Al-Dress, M., Alharbi, T., Alsudairy, R., . . . Alsultan, A. (2016). Clinical characteristics and genetic subtypes of Fanconi anemia in Saudi patients. *Cancer Genet, 209*(4), 171-176. doi:10.1016/j.cancergen.2016.02.003

Levran, O., Erlich, T., Magdalena, N., Gregory, J. J., Batish, S. D., Verlander, P. C., & Auerbach, A. D. (1997). Sequence variation in the Fanconi anemia gene FAA. *Proc Natl Acad Sci U S A, 94*(24), 13051-13056. doi:10.1073/pnas.94.24.13051

Moghadam, A. A., Mahjoubi, F., Reisi, N., & Vosough, P. (2016). Investigation of FANCA gene in Fanconi anaemia patients in Iran. *Indian J Med Res, 143*(2), 184-196. doi:10.4103/0971-5916.180206

Shahid, M., Firasat, S., Satti, H. S., Satti, T. M., Ghafoor, T., Sharif, I., & Afshan, K. (2019). Screening of the FANCA gene mutational hotspots in the Pakistani fanconi anemia patients revealed 19 sequence variations. *Congenit Anom (Kyoto)*. doi:10.1111/cga.12331

Solanki, A., Kumar Selvaa, C., Sheth, F., Radhakrishnan, N., Kalra, M., & Vundinti, B. R. (2017). Characterization of two novel FANCG mutations in Indian Fanconi anemia patients. *Leuk Res, 53*, 50-56. doi:10.1016/j.leukres.2016.11.013

Solanki, A., Mohanty, P., Shukla, P., Rao, A., Ghosh, K., & Vundinti, B. R. (2016). FANCA Gene Mutations with 8 Novel Molecular Changes in Indian Fanconi Anemia Patients. *PLoS One, 11*(1), e0147016. doi:10.1371/journal.pone.0147016

Tamary, H., Bar-Yam, R., Shalmon, L., Rachavi, G., Krostichevsky, M., Elhasid, R., . . . Zaizov, R. (2000). Fanconi anaemia group A (FANCA) mutations in Israeli non-Ashkenazi Jewish patients. *Br J Haematol, 111*(1), 338-343. doi:10.1046/j.1365-2141.2000.02323.x

Wijker, M., Morgan, N. V., Herterich, S., van Berkel, C. G., Tipping, A. J., Gross, H. J., . . . et al. (1999). Heterogeneous spectrum of mutations in the Fanconi anaemia group A gene. *Eur J Hum Genet, 7*(1), 52-59. doi:10.1038/sj.ejhg.5200248

Zareifar, S., Dastsooz, H., Shahriari, M., Faghihi, M. A., Shekarkhar, G., Bordbar, M., . . . Shakibazad, N. (2019). A novel frame-shift deletion in FANCF gene causing autosomal recessive Fanconi anemia: a case report. *BMC Med Genet, 20*(1), 122. doi:10.1186/s12881-019-0855-2
